# Governance tensions in the healthcare sector: a contrasting case study in France

**DOI:** 10.1186/s12913-021-07401-4

**Published:** 2022-01-07

**Authors:** Laurent Mériade, Corinne Rochette

**Affiliations:** University Clermont Auvergne, IAE Clermont Auvergne, CleRMa, Research Chair “Santé et Territoires”, 11 Boulevard Charles de Gaulle, 63 000 Clermont-Ferrand, France

**Keywords:** Governance tensions, Healthcare organisation, Contrasting study

## Abstract

**Background:**

Political and managerial reforms affect the health sector by translating into governance tensions. As identified in the public management literature, these tensions come from the diffusion of management principles and practices from the business world. These tensions manifest at four levels: institutional, organisational, managerial and instrumental. The aim of this research is to understand how these tensions are expressed in healthcare organisations of different status.

**Methods:**

We conduct a contrasting case study exploring the cases of two French healthcare organisations, one private for-profit (clinic) and one public not-for-profit (cancer treatment centre). Our analyses are mainly based on the content analysis of 32 semi-structured interviews conducted with staff (nurses, doctors, management and administrative staff) of these two organisations.

**Results:**

Our results show that these tensions can be distinguished into three categories (tensions on professional values, standards and practices) which are expressed differently depending on the type of healthcare organisation and its main management characteristics.

**Conclusions:**

Unexpectedly, in the for-profit organisation, the most intense tensions concern professional standards, whereas they concern professional practices in the not-for-profit organisation. These analyses can help guide policy makers and healthcare managers to better integrate these tensions into their political and managerial decisions.

**Supplementary Information:**

The online version contains supplementary material available at 10.1186/s12913-021-07401-4.

## Introduction

In OECD countries since the early 1970s, political, administrative and economic reform programmes have been underway and some of them are leading to major changes in implementation of public policies. They are generating particularly acute tensions among the employees and managers of the public services concerned [1, 2] These tensions result from the coexistence of opposing demands, or dualities, such as flexibility and control, differentiation and integration, stability and change [3]. Seen in isolation, each element in tension makes perfect sense, but when both elements appear at the same time, their coexistence may seem illogical. However, the two elements are indivisibly interconnected and interdependent [3].

As current events show, medical and nursing staff working in French health organisations are particularly affected by these tensions [4]. The number of demonstrations, the nature of the demands expressed, the motivations and the violence observed over the last few years reveal the seriousness of these tensions, which have increased over the last few years in French and European public and private healthcare organisations [4]. These tensions have become even more evident in recent months with the global health crisis caused by Covid-19 [5].

The main changes that occurred, enacted in regulations and laws, concern the policy, organisation and functioning of healthcare organisations in France [6]. The increase in the number of patients to care for, the complexity of the cases to be handled due to the ageing of the population, the development of chronic diseases, more and more expensive equipment and an unfavourable medical and nursing demography led to an increase in expenditure, a need to control resources and an overloading of physicians and nursing staff [7].

Hospital reforms have often aimed to improve collaboration and coordination between hospitals as well as access, quality and continuity of care [8]. However, these changes have been accompanied by protests and resistance against the various reforms [9–11], particularly in France, over the last 20 years [4]. The practices that result from these reforms, which according to health professionals are rooted in values from the market sphere [12], sometimes appear incompatible with the public service mission of healthcare organisations [13]. Nunes et al. [3] in analysing their causes, have shown that these incompatibilities are expressed in the form of dualities requiring meta-capabilities to manage them (Plural consensus, orchestrated emergence, distributed connectedness, patient coreness, formalised fluidity, cautious generativness). However, beyond an analysis of their causes, these incompatibilities can also be analysed through their consequences on the personnel and production of health services. Indeed, they have ethical, organisational and managerial consequences [14] that we propose to examine through the prism of governance tensions. This analytical framework, widely tested in public management [15], offers the advantage of accurately identifying the effects of public reforms at four levels (institutional, organisational, managerial and instrumental) [16]. The research question we are addressing here is: in the context of political and managerial reforms in health, are governance tensions expressed in healthcare organisations in any way? Can we identify differences in their manifestation according to the status of the organisation?

We answer this research question in the following way. First, we revisit the importance of tensions in healthcare organisations between the objectives of public reforms and individual objectives. Second, using the analytical framework of public governance tensions [16, 17], we explore the case of two French organisations, one private for-profit (clinic) and one public non-profit (Cancer Treatment Centre). Finally, our results allow us to identify three categories of tensions (on values, norms and professional practices) expressed with different intensities in the two organisations studied. These results guide decision-makers and managers in the health sector towards taking better account of these tensions in their political and managerial decisions.

## Background

### Conceptual framework of the study: Public Governance Tensions (PGTs)

**Fig. 1:**
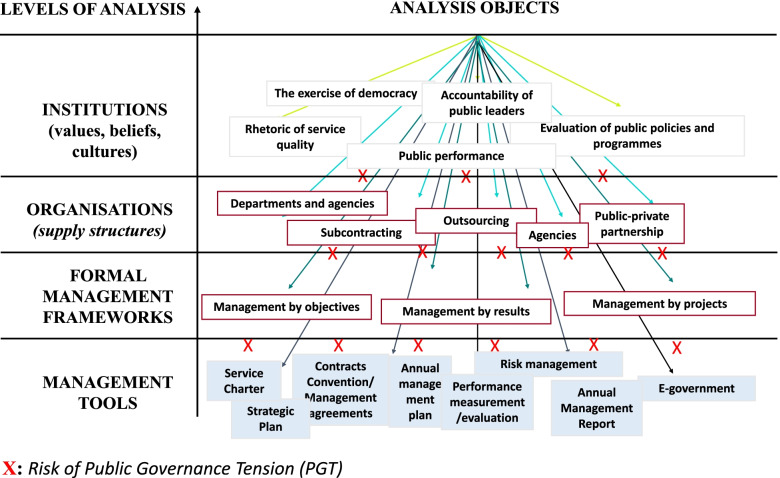
Configuration of a national public governance system (**Source: adapted from **[16]** and **[17]**)**

Conceptual approaches that focus on tensions within national organisations and governance systems offer very interesting analytical potential for the management of public organisations [18, 19] and particularly that of healthcare organisations [20]. Tensions arise from incompatibilities or divergences in orientations and actions between, on the one side, a context shaped by challenges, actor dynamics and specific rules [21] and, on the other side, an organisational environment characterised by its constitutional mission, its governance framework, its structures, its objectives, the status of its staff and the evolution of its management mode [22]. Tensions in healthcare organisations show in particular the importance of creating alliances and joint communication between the micro, meso and macro levels of health systems [23]. Indeed, these tensions are expressed externally between the local and central levels of health systems [24]. They are also expressed internally, as close as possible to the health professionals [9]. Thomson, Murtagh, & Khaw [9] have highlighted the importance of tensions between health policy objectives and the missions of health professionals and now encourage further study of these tensions. Since the late 1990s, reforms applied to public health organisations have led to significant changes in national systems of public governance [2]. These tensions are expressed in particular between national policy priorities and local views and priorities [25]. They create a 'strategic/local' governance boundary that limits the development of multi-level governance of institutions as the health field would probably require [26]. Exploring the effects of reforms on the governance of public organisations, Hudon and Mazouz [17] show that these tensions affect these organisations at four levels: public values, architecture of organisational structures, formal management frameworks and management instruments [16, 17] (Fig. 1). They identify four categories of tensions on the roles, functions and responsibilities of public managers: ethical tensions, organisational tensions, managerial tensions and artefactual tensions (Fig. 1).

- Ethical tensions arise from the difficulty of making the public service mission possible to coexist with increased public performance as considered by the institutional promoters of NPM [27, 28]. It leads to a re-examination of the ethics of staff and public agents [29, 30]. In the health field, these ethical tensions appear in particular in the balance between the individual and collective responsibilities of staff [31].

- Organisational tensions can be caused by the reorganisation of work, the reconfiguration of hierarchical relationships, coordination mechanisms or the allocation of resources [32, 33].

- Managerial tension is a consequence of the evolution of official administrative frameworks (e.g. activity-based financing) in France [34]. It is perceived at the implementation level of the law and administrative rules that lead to tensions between different public professions [35].

- Artefactual tension refers to the instrumental level (or management tools). It arises from the difficulty of matching instruments focused on measuring and evaluating results (the managerial logic) with instruments derived from a bureaucratic logic that focuses on the regulation of rules and means [2, 22, 36]. However, these tensions are not insoluble, and the challenge for public governance is to develop approaches that establish 'virtuous circles' of management instruments from different logics (managerial, bureaucratic or medical) [37].

The interest of applying this framework of analysis of PGTs in health organisations lies in its capacity to illustrate tensions by their consequences, which are often more easily perceived and expressed by health professionals. The application of this framework therefore complements the work that focuses on the causes of tensions [3], with a view to possibly combining these two approaches in future work.

## Methods

The methodology used is that of a contrasting case study. It is consistent with the replicative research strategy recommended by Yin [38]. The operational approach consists of using the same conceptual and theoretical framework to study one case, then another or others. This approach has the advantage of strengthening the external validity of the knowledge produced in a qualitative approach [39]. It is particularly suitable for studying organisational change experienced by healthcare organisations over the last two decades.

### Presentation of the French context and the two cases studied

In France, the healthcare sector has been undergoing major reforms for more than 20 years, resulting in the adoption of a results-based approach [40] inspired by the new public management [41]. Many factors explain the emergence of tensions in France: the adoption of activity-based pricing (T2A), a polarisation and concentration of care activity in urban centres, a large increase in the number of patients to be cared for, more and more often with chronic and complex pathologies, a shift and increased tasks towards care staff and a lower valuation of remuneration, which lead to a lack of nursing staff and recruitment difficulties. Numerous protests and demonstrations have led the Ministry of Solidarity and Health to organise a consultation of the healthcare system's actors in 2020 in order to simplify the organisation and daily life of healthcare professionals in the public sector and to reduce tensions. Among the measures announced from 2021, there is a reduction in the share of activity-based financing (T2A), the abolition of the Inter-ministerial Committee for the Performance and Modernisation of the Hospital Care System, which will be replaced by a French National Investment Council, a revaluation of public hospital staff and the opening of discussions to the private health sector.

In France, there are three types of healthcare organisations:

- Public health organisations (1,389 organisations in 2019 [42]). These are legal persons under public law with administrative and financial autonomy. They are subject to State control. Their main vocation is neither industrial nor commercial. These university hospital centres are involved in both medical and pharmaceutical research and medical and paramedical education.
- Private not-for-profit establishments (689 organisations in 2019), generally from religious, charitable or mutualist movements. These are private health establishments. They are qualified as being of collective interest as long as they provide a public hospital service.
- Private for-profit organisations (1,014 organisations in 2019). These are private clinics with a health and commercial vocation managed by private funds and management. Nevertheless, they have a close link with public reforms and policies, since Public Social Security reimbursements represent more than 60% of their revenues in 2020 [36].

We have chosen two cases to reflect the particularities of French private health organisations, a clinic and a cancer treatment centre (in French *Centre de Lutte Contre le Cancer*—CLCC), both located in the same region of France (Table 1).

**Table 1:** Two contrasting cases: CLCC (public mission) and clinic (private for-profit)

**Contextual and organisational factors**	**CLCC**	**CLINIC**
Status	Public, but close to public structures such as university hospitals (care, research and teaching missions)	Private care mission
Connection entity	Member establishment of *Unicancer* federation of 20 establishments	Establishment of the *Elsan* group which is the leading operator of private clinics in France
Location	Conurbation of of more than 290,000 inhabitants	Town of 25,000 inhabits
Organisational specifity	Specialist in the management of cancerous pathologies Model of care for cancer patients	Multi-specialty community medicine and surgery
Number of related professionals	724 staff members including 102 doctors	212 staff members: 62 doctors and 150 non-medical staff (care staff, administrative staff, etc.)
Annual number of patients treated	32,000	8000

The choice of these two establishments is dictated by contrasting contextual and organisational considerations. These two structures are private and operate in the same health context (public mission but pursuing different profit and not-for-profit objectives). One is located in a major city, which can facilitate the professional mobility of the staff of these organisations. The staff have many public and private health establishments nearby to carry out their activities, unlike the smaller city in which the clinic is located. This one is characterised by a more limited supply of health services, which is a limit to the professional mobility of staff in this area. The two organisations are 70 kms apart.

## Method of data collection

### Design and recruitment

We adopt for this study a phenomenological approach to explore and describe views of the professionals of healthcare organisations. Phenomenology focuses on how individuals perceive the world [43] in our case how they perceive tensions in healthcare organisations. This method is increasingly used to access the representations of health professionals or patients [44, 45]. We aimed to understand the perceptions and awareness of tensions that affect organisational functioning in two different organisational contexts.

We conducted a series of semi-structured individual interviews with 32 healthcare personnel from two French hospitals of different status (clinic and CLCC). All methods were carried out in accordance with relevant guidelines and regulations. The interviews are part of a written research process built in strict compliance with the rules of the ethical charter of the university research laboratory of the authors of the study. The interviews were conducted with the agreement of the management of the two organisations studied and approved by their boards of directors. The experimental protocol used was brought to the attention of the ethics committees of the two organisations studied. The selection of interviewees was made on a voluntary basis from a mailing designed and sent by the management of the two organisations studied. The e-mail sent specified the objective of the study, the modalities of the interview (anonymity, recording, use of the collected data, names and status of the interviewers, duration and location). Interested persons who agreed to be interviewed were invited to contact the researchers by e-mail or telephone to schedule the interview. Our research is a non-interventional research so it is not subject to the rules of the French Jardé law (law n° 2012–300, 2012 March 5th). This category of research based on the collection of individual representations doesn't need a written consent. However, before collecting the discourse of our interviewees for each of them we have detailed our research objective and methodology to obtain and audio-record oral informed consent from each respondent.

Three main categories of staff were investigated: doctors, nursing staff and staff with managerial functions (coordination, steering, administrative) (Table 2).

**Table 2:** Respondents in the two organisations

Clinic			CLCC		
Code	Function	Duration (min)	Code	Function	Duration(min)
Ia1	Care Assistant	42	Ib1	Director of Care	65
Ia2	Human Resources Manager	42	Ib2	Medical assistant	36
Ia3	Financial Manager	39	Ib3	Intensive Care Nurse	28
Ia4	Pharmacist	31	Ib4	Nurse	39
Ia5	Coordinating Officer	44	Ib5	Emergency Doctor	54
Ia6	Doctor	34	Ib6	Medical Secretary	39
Ia7	Anaesthesiologist	54	Ib7	Administrative logistical Manager	42
Ia8	Hospital Services Agent	32	Ib8	Medical Assistant	44
Ia9	Healthcare Manager	27	Ib9	Medical Secretary	37
Ia10	Care Assistant	29	Ib10	Surgeon	48
Ia11	Quality Engineer	44	Ib11	Computer Scientist	57
Ia12	Care Assistant	45	Ib12	Senior Health Executive	57
Ia13	Coordinating Manager	39	Ib13	Imaging Department Manager	51
Ia14	Executive secretary	33	Ib14	Deputy Director	73
Ia15	Nurse	24	Ib15	Doctor	38
Ia16	Nurse (surgery)	29	Ib16	Financial Affairs Director	39

### Data collection

Our sample of 32 peoples consisted of 16 professionals from each of the two organisations studied. We conducted semi-structured in-depth interviews at the hospitals (CLCC and clinic location). The information generated from these interviews was intended to enhance our understanding and knowledge about governance tensions. Each interview lasted for 24 to 73 min. We began the semi-structured in-depth interviews by asking general questions and gradually introducing more specific questions about their values, perceived and felt tensions and the meaning given to their work. The interview guide is structured around four themes: the work environment and its evolution, the organisational, professional and personal values, the tensions felt or perceived, and the meaning given to work (see additional file). After obtaining the consent of each interviewee for the recording and transcription of their speech, we transcribed and analysed the 1,293 min of recorded interviews.

### Method of data coding

Based on the conceptual framework of PGTs [16, 17], we constructed an analysis grid adapted to our research question. This allowed us to code our materials (Table 3) in order to identify and characterise the tensions of public governance present in the two organisations studied.

**Table 3:** Interview coding grid

**Levels of governance** (Mazouz et al., 2012)	**Governance tensions** (Mazouz et al., 2012. Hudon and Mazouz, 2014)
**Institutions** (values, beliefs, cultures)	**Ethical tensions** (in terms of values and standards)
**Organisatons** (supply structures)	**Organisational tensions** (at the level of work organisation and coordination mechanisms)
**Official management frameworks** (laws, rules, procedure)	**Managerial Tensions** (in the application of laws, rules and procedures)
**Management tools** (HRM, accounting, logistics tools, etc.)	**Artifactual tensions** (at the level of management instruments)

## Results

Our results highlight the presence of governance tensions at four levels. However, these tensions manifest differently depending on the characteristics of the healthcare organisations.

### Ethical tensions and changing institutional values

In the two establishments studied, we identify ethical tensions linked to the questioning of the weight of financial constraints, economic viability and compatibility with the public care mission. The staff are aware of their representations, which are strongly anchored in the values of care. These values are at the very heart of the health professions. Thus, achieving a different meaning requires deconstructing the representations according to which good care is not compatible with a strong focus on the financial dimension.

*"We have common representations, common values such as "yes, we provide better care in the public sector than in the private sector where profitability and medicine do not mix*" Ib11.

These representations largely explain the ethical tensions felt when the term profitability is introduced in the managers' speeches and activity reports. Nevertheless, these ethical tensions are expressed somewhat differently for CLCC staff and clinic staff.

In the clinic, the financial dimension is an important issue and a question of the sustainability of its activity, but it is rather well accepted. Managers explain the need to reconcile care and financial performance to ensure the clinic's survival. The staff integrate these values while ensuring their compatibility with the public mission of "care".

*"We are asked to do this or that, to review the way we work with patients*. *I think it's essential to have these management tools to be able to explain to the teams why we do this. We can't say that we have pressure on the results, but we are aware of what is at stake and what the clinic has to do to exist. "*Ia9

The professional practices and behaviours of the staff make it possible to reconcile the imperatives of patient care with a rational use of resources.

*"I am quite flexible as long as their work is done, … mutual trust. "*Ia3

For the clinic's physicians, *"profitability depends on the best possible quality of care. It is not an end in itself*" Ib7.

At the CLCC, the values of care prevail over managerial values in this construction. Practices and behaviours are part of a setting where economic performance is very present, but in the minds of the interviewees, the values of the public service mission predominate.

*"The advantage of a CLCC is that the management is entrusted to a doctor, unlike a university hospital*. *Production, which is care, is then at the origin of the strategic thinking, how to provide better care and not exclusively, how to increase the volume of activity.* "Ib17.

Although the idea of profitability is very much present in the CLCCs' general management, for them, profitability must be at the service of patients and aims to maintain and perpetuate the medical and care offer.

*"The public service character is linked to the fact that we are financed by public money*. *So in this context, because of our mission of collective interest, the profit must not be excessive*" Ib14.

Thus, at the CLCC, the responses we obtained clearly highlight the values of care as unquestionably dominating those of management.

### Organisational tensions generated by the diversity of professional standards

The staff of both establishments report a significant change in working practices.

In the care professions, managers and nursing staff note an increasing emphasis on "technical care" to the detriment of "relational care". They seem to regret it but they understand it.

*"We are arriving at a new type of care which, in my opinion, has become much more technical, standardised, classified and normalised.”*Ib13

Professional competence is now anchored in a technical skill that is guided by the application of rules and procedures that some find comfortable. These provide a reassuring framework for the exercise, it is that of correct technical gestures and of the control associated with them.

*"Confidence is built on technical skills but also on the attitude we have towards the patient*. *The patient needs rigour and humanity*" Ib1.

In the clinic, the professional standards that guide the activity of each category of staff are generally accepted. The compatibility of personal values with professional standards depends on the acceptance of the system and the tools for steering the activity.

*"Management tools make it easier to accept... heads of department are now able to anticipate whether they are actually going to replace a person or not... they say to themselves 'I'm going to have heavy patients now, I absolutely have to take on an extra person without being afraid of being reprimanded because there is a* justification'. "Ia2

In this constrained framework (compliance with administrative-financial standards), the respondents underline the challenge they face in reconciling profitability with the best possible care of the patient.

*"Yes, profitability is a concern, there may be a temptation to buy cheaper medical devices, but you have to keep in mind that it is the patients who are behind it. Balancing the two is a challenge for me*. "Ia4

It seems that all the staff internalise this meaning. Nevertheless, for some of the interviewees, their work is more a manifestation of submission to authority than a true adherence to this meaning.

*"The management makes us feel that we have to be profitable, whereas for us the human side is more important. It's true that there are tensions at that level."*Ia1

For both hospitals, professionalism is expressed in a high level of demands on patients. This is particularly prevalent in discourses for the CLCC where care is particularly complex. This very strong requirement expresses a very pronounced professional commitment.

*We have to be careful to live up to our reputation, not to disappoint*. "Ib4.

*"You don't become a carer like you become a mechanic, you have to take care of your patients, you have to relieve them, you are a carer*" Ib4.

The regulation of organisational tensions is achieved through a stronger focus on professional practices and on patient care rather than on technical standards, although these remain important.

### Managerial tensions between practices and professional standards

Within healthcare organisations, cooperation with other professionals and organisations is seen as helping to reduce the risk of tensions on professional practices that the managerial practices of the NPM [34] may cause.

However, managerial tensions may persist because, according to the interviewees, in a context of competition, private and public health organisations are seeking to gain patients and market share. This situation leads to limited collaborations that most of them regret.

*"We are in direct opposition to the public and private sectors, but we defend the patient's pathway. In all cases, the patient goes to the clinic, to the university hospital, comes back to the CLCC." Ib1*

In the face of managerial tensions between intra- and inter-organisational professional practices, teamwork takes on its full meaning.

This is particularly true in the CLCC, because the team plays the role of regulating professional tension by making it possible to objectify a situation and thus to restore value and meaning at work.

*"In the oncology sector, there are very strong friendships that develop because there are difficult situations to live through and this is part of the life of the organisations. There are human stories. "« … ». The team is a valve when we are experiencing a difficult situation in our workplace, the informal exchange times we used to have with the other people in the team allowed us to regulate, but today, the reorganisation of the work has reduced these spaces that allowed us to talk about our ethical questions*" Ib1.

Cooperation with the patient also makes it possible to align the practices of the CLCC's health professionals. The attention paid to the patient as a person and his or her well-being unites the professionals despite the tensions.

*"Professionals who remain on the "I know" perspective for the patient and others who have understood that the patient has much to teach us*" Ib1.

On the other hand, for the professionals in the clinic, cooperation is not spontaneously mentioned as a deliberate practice. It is rather a response to a normative injunction, to a standard that is prescribed by the management. The staff mentioned the difficulty of cooperating with other structures, the reason being the existence of a form of hidden competition (capturing more patients than the "competitor"). This posture nevertheless questions the health and care professionals who are more naturally inclined to cooperate for the benefit of the patient.

*"Cooperating would have made sense for all of us*. *They refused, they were afraid. We will still manage without them, but it's sad".* Ia4*.*

### Instrumental tensions modifying professional practices and behaviour

Many professional practices of healthcare staff have had to change as a result of the reforms that have affected healthcare establishments in France since the early 2000s (introduction of activity-based pricing in 2004, the HPST law in 2009, "*Ma santé 2022*", a law enacted in 2019). These reforms have largely contributed to the development of management tools and indicators in the daily work in all its aspects of health personnel (hospital staff, nurses, care assistants, managers, physicians) [46].

In the clinic studied, managers use these indicators and management tools to translate management's orientations to the healthcare teams in order to reduce tensions between professional practices and management standards.

*"You have to be profitable despite everything, while ensuring quality. The quality of care is paramount. But the staffing indicators in my department also allow me to say to management, below that, you can't do less, we won't go below that staffing level, it's not possible*" Ia5.

Within the CLCC, there is a great need to understand the meaning of management tools and indicators. There is a real questioning about the relevance of the tool but also over its ability to report on situations.

*"We have to change our indicators completely because they are obsolete, we have to adapt them to changes*. *I am told that there is an increase in expenditure in my department, whereas we have played the game and made savings. I am being charged for expenses that are made by the anaesthetists and over which I have no control. "*Ib9.

It is up to the CLCC managers to deal with the tensions generated by the financial dimension, which is becoming more and more prevalent. It is their challenge to reconcile management tools with the professional practices of medical and nursing staff. This is a delicate exercise that causes for some of them discomfort, but it is accepted and generally perceived as a necessary challenge to be met.

*"We need to increase activity, when I say that to the nurses, it's still disturbing*. *But I'm fully aware that I have to talk about money in my job. But it's also a challenge to my way of looking at things*" Ib4.

## Discussion

### Three forms of tension, mainly professional

The health governance literature is gradually showing that in health organisations, professionals navigate tensions between their discretionary space, the parameters set by a central decision-maker, and their quest for integration and differentiation [47]. Our results show that the tensions of public governance present [16, 17] in healthcare organisations [9, 22] are expressed in a distinct way depending on the governance mode of the organisations. To our knowledge, this has not been demonstrated in a very illustrative way until now. Moreover, our results show that it is not only protests and resistance [9] that emerge from the discourses, but the capacity of the staff to manage these tensions and to make them liveable, even acceptable [48].

The tensions observed concern the four levels of governance (institutional, organisational, managerial and instrumental). The responses made by staff to manage them are strongly influenced by the context in which their organisation operates (Table 4). Our study highlights three main categories of governance tensions that are expressed primarily at a professional level in healthcare organisations: tensions concerning professional values, standards and practices (Table 4).

**Table 4:** Governance tensions in healthcare organisations

Levels of governance	Clinic		CLCC	
	**Governance tensions**	**Responses to tensions**	**Governance tensions**	**Responses to tensions**
**Institutional**	Tensions on professional practices	- Translating steering tools	Tensions on professional practices	- Role of middle management
	Tensions on professional standards	- Acceptance of the search for profitability	Tensions on professional values	- Role of professional beliefs
**Organisational**	Tensions on professional standards	- Role of the patient pathway	Tensions on professional practices	- Enhanced cooperation between professionals
**Managerial**	Tensions on professional values	- Professional beliefs focused on patient care	Tensions on professional values	- Staff sacrifice - Staff commitment to the care profession
	Tensions on professional standards	- Cooperation and teamwork		
**Instrumental**	Tensions on professional standards	- Role of wage increases - Role of management indicators	Tensions on professional practices	- Appropriation of management tools - Cooperation

First, the tensions on professional values are mainly described by staff as tensions between care values and financial values. These tensions observed in the two hospitals studied, come from the difficult reconciliation of financial values, particularly those resulting from the hospital reforms of the last 20 to 30 years, with the traditional values of care carried by the health professionals. Tensions regarding professional standards stem mainly from the difficult reconciliation between the technical and relational dimensions of staff. This category of tensions results from the evolution of medical and nursing work towards technical dimensions to the detriment of its relational dimensions between health professionals or with patients. Lastly, tensions on professional practices arise from the difficulty for staff to reconcile the care functions with the administrative functions brought about by the new management instruments. These tensions stem from the development of management tools in healthcare organisations (generalisation of activity-based pricing, development of cost calculation tools, reporting to regional health agencies). These are gradually replacing the managerial practices that until now have been mainly oriented towards the management and steering of care functions (Table 4).

However, secondly, these three categories of tension are expressed differently depending on the type of organisation and, in particular, its main management characteristics.

In the private for-profit clinic, contrary to all expectations, the analysis of the verbatim reports reveals that tensions related to professional standards are the most important (Table 4). In the interviews, staff emphasised the pre-eminence of technical work standards and procedures in their work and the delicate reconciliation with the relational dimension of their work. This is evidence that these tensions are present and that they guide the ways in which staff act. These tensions are expressed at four levels of tension (institutional, organisational, managerial and instrumental) and the clinic staff respond to these tensions in the following ways:

-an acceptance of the search for profitability in order to perpetuate the clinic and its place in the region,

- increased professional cooperation and teamwork,

- greater negotiation with the management of the establishment with regard to remuneration and the definition of management indicators (Table 4).

The staff of the establishment mentioned tensions concerning professional values and practices, but in a much less marked way. They indicate that these tensions are reduced, on the one hand, by a greater translation of steering tools by middle managers and, on the other hand, by the mobilisation of staff's professional beliefs focused on patient care (Table 4).

On the contrary, in the not-for-profit public service CLCC, it is mainly tensions on professional practices and, to a lesser extent, tensions over professional values that the staff are required to respond to (Table 4). For these staff, the main tensions arise from the difficult cohabitation between the management tools accompanying public reforms in French healthcare organisations and the care functions attached to the profession of care provider. To a lesser degree, related tensions are expressed between the care values of the staff and the financial values of the reforms. Nevertheless, for the staff of this non-profit organisation, these tensions on professional practices and values can be managed and reduced by:

- middle management,
- the professional convictions of health workers and their personal commitment,
- cooperation between professionals inside and outside the organisation to provide the technical and relational functions of care,
- The appropriation of management tools in the professional practices of staff (Table 4).

Consequently, the identification of these three forms of tension and their modes of expression in two types of health establishments makes it possible to understand and anticipate the forms of appropriation of health policies in care organisations.

In conceptual terms, by analysing the consequences of tensions at four levels (institutional, organisational, managerial and instrumental) (Table 4), the conceptual framework mobilised and our analytical work make it possible to identify tensions arising from the activity of health professionals. By studying the consequences of tensions on professional activity, this analysis completes the one carried out on the causes of tensions [3] which focuses on the managerial tensions of a specific activity (quality management, information management, financial management, etc.). It shows that alongside the tensions caused by each managerial "object", there are also more systemic tensions generated by the overall governance of the health system and the political reforms that are particularly important in France.

In practical terms, national policy makers and managers in organisations need to identify and discuss good practice in order to design and implement successful health reforms [7]. Our results demonstrate the need to also integrate an analysis of the risks of professional tensions related to political and managerial decisions in order to build health policies adapted to their implementation context.

### Different intensities of professional tensions according to the type of healthcare organisation

From a theoretical but also practical point of view, this study allows us to propose a precise distinction between the forms and intensity of governance tensions in healthcare organisations (Table 5).

**Table 5:** Forms and intensity of professional tensions in healthcare organisations

**Intensity of tensions**	**Private for-profit healthcare organisation**	**Public not-for-profit healthcare organisation**
Strong	**Tensions on professional standards** (between technical and relational dimensions of work)	**Tensions on professional practices** (between management instrumentation and care functions)
Moderate	**-Tensions on professional values** (between care values and financial values) **-Tensions on professional practices** (between management instrumentation and care functions)	**Tensions on professional values** (between care values and financial values)

Against all expectations, in the for-profit organisation, which is apparently a less bureaucratic organisation, it appears paradoxically that it is the professional standards (procedures and work standards) that can be the most disruptive for the staff (Table 5). Performance objectives (economic or service quality) and reporting procedures come into strong tension with the relational dimensions of care functions. However, it is also in the face of these tensions that health professionals find the most adaptation, notably by orienting these standards towards patient care. The resolution or at least the reduction of these tensions is more regularly sought through the construction of new professional standards articulated mainly around the patient and his/her care.

Tensions on professional values and practices also exist in these organisations but are more moderate. They seem to stem from professional tensions over standards, especially when they require a practical or value-based translation of prescribed procedures.

In the not-for-profit organisation, which might on the contrary appear more bureaucratic, it is the professional practices that are the source of tensions, particularly when they neglect the relational dimensions of care work (Table 5). These tensions in professional practices are most clearly linked to the difficult reconciliation between the technical dimensions of care work (medical and organisational dimensions of care) and the relational dimensions. The resolution or at least the reduction of these tensions is sought through greater intra- and inter-organisational cooperation between health professionals. In this case, public reforms or managerial decisions concerning the necessary improvement of medical and organisational practices will be more easily prescribed by encouraging staff cooperation.

Tensions on values also exist but are less intense. They are often managed by a greater investment of staff and a gradual appropriation of the financial values of care by the values of care.

This distinction between the intensity of these three tensions in the two most traditional forms of healthcare organisation (for-profit and not-for-profit) begins to define a field of research into the effects of modes of governance on the expression of tensions that has so far been little explored in the health literature. Indeed, while the literature has shown in detail that tensions in organisations can arise from the coexistence of different 'modes' of governance (generally characterised by markets, hierarchies or networks) [47], little work has so far shown how these tensions are expressed and how the mode of governance can regulate their intensities.

This research focuses on the tensions of intra-organisational public governance, highlighting in particular the tensions generated by the decoupling between the orientations largely stemming from a logic of new public management carried by politicians and the capacities of managers in charge of public service missions who have to comply with the rules of good management and principles enacted by politicians [48]. A solution can be found in the literature on meta-governance to ease the tensions. This is based on the ability to involve all actors in the definition of a policy agenda and of alternative options (network participation) and by reducing destructive tensions through the acceptance of a flexible adjustment of ends and means (network management) [48].

Also, from a managerial point of view, this distinction in the intensity of tensions, for the first time, also offers managers and public policy makers the possibility to better understand or anticipate how their reforms or management decisions will influence the work of health professionals.

### Contributions

This work on the modes of expression of governance tensions in healthcare organisations of different status presents three contributions, theoretical, methodological and managerial.

First, at the theoretical level, this study shows that these tensions are expressed mainly at the professional level, in three distinct areas: professional values, professional standards and professional practices. This result complements work on the tensions caused by public policy changes in the health sector [9, 20, 24]. It shows, on the one hand, that in this sector, these tensions are expressed in organisations mainly at the professional level and, on the other hand, that in addition to the analysis of their causes [3], these tensions are also observable through their consequences on the personnel. At the same time, this study also indicates that, in the private for-profit clinic, contrary to all expectations, tensions around professional standards appear with much greater intensity than those relating to professional values or practices. On the contrary, in the not-for-profit public service CLCC, it is mainly tensions relating to professional practices that dominate and to a lesser extent those relating to values.

Secondly, from a methodological point of view, this study has made it possible to construct an initial research method using the contrasting case study to study governance tensions in healthcare organisations. This method can be replicated and tested in future studies, particularly by studying other forms of contrast.

Finally, and thirdly, from a managerial point of view, the identification of these three categories of tensions in healthcare organisations offers health policy makers and healthcare organisation managers the opportunity to identify how tensions develop in healthcare organisations and are managed by staff and managers. Addressing these tensions in the design and implementation of health policies is an important issue.

In this respect, the responses given to these tensions by the managers and staff of the establishments studied (the role of cooperation, the values and beliefs of healthcare staff, the patient pathways, the translation of management tools by managers—Table 4) represent an intellectual capital [49, 50] that is probably ignored by the leaders of these establishments, but which this study makes it possible to begin to identify as potential tools for the management of tensions in healthcare organisations.

### Limitations and future research

A first limitation of this study is related to the lack of exhaustiveness of our approach, which is limited to the study of two French healthcare organisations and does not allow us to take into account all the contextual specificities of governance tensions. A future step in this work is to carry out other comparative studies to support and enrich the categorisation of tensions proposed in this study. A second methodological limitation concerns the selection of interviewees, which was done on a voluntary basis. Future studies will have to determine whether selection by sampling or randomisation could modify the results obtained.

## Conclusion

The purpose of this article was to better understand how governance tensions are expressed in healthcare organisations. The contrasting study of two healthcare organisations, one private and for-profit, the other public service and not-for-profit, allowed us to identify more precisely the characteristics of these tensions. This is a significant improvement in our knowledge of these tensions in healthcare organisations and how they are constructed. Future work already in progress should enable us, on the one hand, to confirm the categorisation of tensions put forward but also, on the other hand, to justify by other explanations their different modes of expression according to healthcare organisations.

## Supplementary Information

**Additional file 1.** Interview grid The interviewee's situation 1.The work environment 1.1. The functioning of the organization 1.2. The management 2. About tensions 3. The question of Values 4. Meaning of work

## Data Availability

The datasets used and/or analysed during the current study available from the corresponding author on reasonable request.
