# Supplementary material for: Governance tensions in the healthcare sector: a contrasting case study in France
Source: BMC Health Serv Res. 2022 Jan 7;22:39. doi: 10.1186/s12913-021-07401-4 (PMC8739355; doi:10.1186/s12913-021-07401-4)
Supplement: Supplementary file 1 — Additional file 1. Interview grid The interviewee's situation 1.The work environment 1.1. The functioning of the organization 1.2. The management 2. About tensions 3. The question of Values 4. Meaning of work [file 12913_2021_7401_MOESM1_ESM.docx]

**Additional File**

**Interview grid**

Introduction: Reminder of the context and objectives of the study, of the use made of the data, confirmation by the interviewee of his or her agreement to participate and his or her non-objection to the recording.

**The interviewee's situation**

- Can you tell us about your training, professional background, and how long you have been working in this organization?

- What are your current and past functions in this organization: position, discipline, scope of your activities, responsibility, location of professional activity and work context?

**1.The work environment**

**1.1. The functioning of the organization**

- Can you tell us about the objectives of a health care institution and more specifically those of your institution? What do you think of the context in which healthcare institutions, and your institution, evolve?

- Can you speak to us about the means to achieve these objectives and how these objectives are evaluated?

- How would you assess your institution in relation to others and the way it operates?

**1.2. The management**

- Can you tell us about the work organization in your establishment, your department, your team? Rules, procedures, coordination mechanisms?

- What would you say about the evolution of work methods? Do you use new tools or instruments? What do you think are the reasons for these new ways of doing things? How do you perceive these evolutions?

- Can you describe the links and relationships you have with other staff (directors, administration, logistics, nurses, care assistants, etc.)? With your superiors, your colleagues?

- What is the place of trust?

- What do you think of the relevance and effectiveness of the management tools used by the directors or managers of your institution?

**2. About tensions**

Are there any tensions or disagreements between your opinion of the management of the institution and the way in which the leaders manage ?

Are there tensions with your superiors and/or colleagues? What are the causes (management and evaluation tools used, management style, etc.)? How can they be overcome?

**3. The question of Values**

What can you tell us about your organization's values and your own?

- What are these values ? What can you tell us about their evolution?

- How do they manifest themselves in the organization?

What are the values specific to your work (public, private, managerial values)? Are there conflicts of values or tensions related to principles or work rules in your organization or department? If so, do you manage to overcome these value conflicts and how?

Are there conflicts of values or tensions related to principles or work rules among the staff of your service?

**4. Meaning of work**

What can you tell us about your involvement in the work? What place does your work take?

What sense do you give to your work? Do you see it changing?

What can you tell us about the organizational culture?
